# Association between cardiovascular diseases and dementia among various age groups: a population-based cohort study in older adults

**DOI:** 10.1038/s41598-023-42071-8

**Published:** 2023-09-09

**Authors:** Laurie-Anne Boivin-Proulx, Judith Brouillette, Marc Dorais, Sylvie Perreault

**Affiliations:** 1 Department of Cardiology, Faculty of Medicine, University of Ottawa Heart Institute, Ottawa, ON Canada; 2 Department of Psychiatry and Addictology, Université de Montréal, Montreal, QC Canada; 3 StatSciences Inc., Notre-Dame-de-l’Île-Perrot, QC Canada; 4 Faculté de Pharmacie, Université de Montréal, Succursale Centre-Ville, Case Postale 6128, Montreal, QC H3C 3J7 Canada; 5 Centre de Recherche en Santé Publique (CReSP), Partenaire CIUSSS du Centre-Sud-de-l’Île-de-Montréal et l’Université de Montréal, Montreal, QC Canada

**Keywords:** Cardiovascular diseases, Dementia

## Abstract

The link between cardiovascular (CV) risk factors or diseases and dementia is documented. There is conflicting evidence whether age moderates the association. We need to study this gap so that research and clinical initiatives target appropriate age groups. A cohort of 320,630 adult patients without dementia was built using Quebec healthcare databases (1998–2010). The CV risk factors were hypertension, diabetes and dyslipidemia, while diseases included stroke, myocardial infarction (MI), chronic heart failure (HF), and atrial fibrillation (AF). Dementia risk and CV risk factors or diseases were assessed using incidence rate ratios and Cox regression across age groups. The cohort presented by mainly female sex (67.7%) and mean age of 74.1 years. Incident rate of dementia increased with age, ranging from 4.1 to 93.5 per 1000 person-years. Diabetes, stroke, HF and AF were significantly associated with dementia risk, hazard ratios ranged from 1.08 to 3.54. The strength of association decreased in advanced age for diabetes, stroke and HF. The results suggest that prevention of diabetes, stroke, HF and AF are crucial to mitigate dementia risk. The pathophysiology of dementia in younger and older populations seems to differ, with less impact of CV risk factors in advanced age.

## Introduction

Dementia is a major public health care concern, with 46.8 million incident cases worldwide in 2015 and numbers nearly doubling every 20 years^1^. The worldwide economic costs due to dementia have been estimated at 818 billion dollars in 2015, and the threshold of 1 trillion dollars was likely crossed in 2018^1^. Dementia accounted for 10 million disability-adjusted life years among the elderly in 2010 and is estimated to increase by 86% by 2030^2^. However, no effective strategies are currently available for treating dementia but according to a meta-analysis of randomized clinical trials, donepezil, rivastigmine and galantamine may delayed the associated cognitive decline^3–5^.

Cardiovascular (CV) and neurological disorders frequently co-exist due to shared risk factors, bidirectional interactions and potentially similar underlying pathophysiological processes^6^. Indeed, both diseases are related to ageing, which causes macro- and micro-vascular dysfunction, ischemia, infarction, inflammation, senescence and fibrosis^7–9^. CV risks factors, such as diabetes mellitus and hypertension, are modifiable risk factors of dementia, and it has been suggested that a one quarter to a third of dementia cases could be prevented if modifiable risk factors were eliminated^10–15^. While neuropathological studies have shown that at an advanced age, both neurodegeneration and cerebrovascular disease contribute to the cognitive impairment^16–18^, other studies have shown that the dementia risk associated with CV risk factors decreases with age^19,20^. Conflicting evidence remains poorly explained and large population-based studies are needed to clarify the influence of age on the association between CV risk factors or diseases and dementia. We used a cohort study to assess the association between CV risk factors or diseases and the incidence of dementia in different groups of age.

## Methods

### Data source and ethics declarations

Population-based cohorts were built^21–24^ using the Med-Echo administrative databases (hospital discharge reports), the *Régie de l’assurance maladie du Québec* (RAMQ) medical services, and RAMQ public drug plans all databases administered by the RAMQ^25,26^. The databases were linked via encrypted health insurance numbers. Combining the information from Med-Echo databases, RAMQ medical services, and RAMQ drug plan databases, we obtained a comprehensive history of health care services. As systematic assessments of cognitive impairment are limited in population-based cohorts, validated cognitive outcomes captured at the population-level are restricted to medical diagnoses of Alzheimer's disease and other major neurocognitive disorders (dementia), and/or prescription of a medication related to dementia^27^.

The databases were linked through encrypted personal health numbers. We have access to RAMQ administrative database, where no patient or physician identifiers were provided to the researchers; only scrambled identifiers were used throughout the study. Data access requests for the RAMQ databases are approved by the *Commission d’accès à l’information du Québec*. All efforts were made to maintain the data confidentiality. Information from these databases provided a comprehensive picture of hospitalizations. The protocol received the approval of the University of Montreal Ethics Committee appointed *Comité d’éthique de la recherche en sciences et en santé* and all methods were performed in accordance with the relevant guidelines and regulations. The RAMQ covers all Quebec residents for the cost of physician visits, hospitalizations and procedures, and 94% of Quebec citizens aged 65 and older for the drug insurance plan^28^.

### Study population and design

The cohort was based on a random sample of 40% of the total cohort of patients aged 66 years and above between January 1998 and December 2010 in the province of Quebec, Canada. The follow-up of the total cohort of patients ended in December 2016. We created six age groups: 66–69 years, 70–74 years, 75–79 years, 80–84 years, 85–89 years, ≥ 90 years. Each patient was included in the age group when he reached the corresponding age. Thus, a patient may be included in more than one age group during the follow-up. For each age group, patients who had a diagnosis of dementia (International Classification of Diseases, 9th Revision (ICD-9): 46.1, 331.0, 331.1, 331.5, 290, 294; ICD-10: G30, F00–F03) and/or was prescribed a medication associated to dementia (donepezil, rivastigmine, galantamine, memantine) within the five years prior to the age group entry were excluded.

### Assessment of dementia

The primary outcome was the incidence of dementia between entry and exit within each age group during a follow-up of 5 years. The incidence of dementia was defined as follows: a diagnosis (primary or secondary) of dementia during a hospitalization (ICD-9: 46.1, 331.0, 331.1, 331.5, 290, 294; ICD-10: G30, F00–F03), and/or two diagnoses of dementia (ICD-9: 46.1, 331.0, 331.1, 331.5, 290, 294; ICD-10: G30, F00–F03) within two years in the medical services database, and/or prescription of a medication related to dementia (donepezil, rivastigmine, galantamine, memantine) among patients living in the community. The accuracy of the definition of dementia using the medical records of the patients and/or the use of medication shown a specificity of 98.7%, and a predictive positive value of 74.0%^27,29^. The identification of dementia case with only medication ranged between 12.0 and 32.5% according to the age groups.

### CV risk factors or diseases

Association with incident dementia was studied for the following CV risk factors or diseases diagnosed within the five years preceding the age group entry using ICD-9 or ICD-10 (Table S1): hypertension, diabetes, dyslipidemia, stroke, myocardial infarction (MI), chronic heart failure (HF) and atrial fibrillation (AF).

### Statistical analyses

Descriptive statistics were used to summarize the demographic and clinical characteristics of patients. Incident rates ratios (IRR) [per 1000 persons-year (PY)] of dementia per CV risk factors or diseases, and per age group were calculated. The association between incident dementia and all CV risk factors or diseases in each age group was assessed using a Cox regression model by CV risk factors or diseases adjusted for age and sex, and also with a multivariate Cox regression model. Patients were censored at the time of enrolment in a non-governmental drug coverage plan, admission to a long-term care facility, or the occurrence of the primary endpoint (dementia) or death between the date of entry and the 5-year of follow-up (whichever occurred first). The patient’s censored status was updated every 30 days. The proportional hazards assumption was checked using the Schoenfeld residuals test. Variables included in the multivariate Cox regression were age, sex, hypertension, diabetes mellitus, dyslipidemia, having a prior event of stroke, MI, a diagnosis of HF or AF. Cox regressions were also done by adding other covariates such as, major bleeding, systemic embolism, peripheral vascular disease, and chronic kidney disease using ICD-9 or ICD-10 codes (Table S1). Hazard ratios (HR) with 95% confidence interval (CI) were calculated. And as a secondary analysis, Fine and Gray regression models were used to adjust for death as a competing risk^30^. All analyses were performed using SAS 9.4 statistical software (SAS Institute, Cary, North Carolina).

### Consent to participate

No informed consent was needed for this study. This research was based on information extracted from user files without interactions with the research participants in accordance with the provisions of Section 125 of the Act respecting access to documents held by public bodies and the protection of personal information (L.R.Q c. A-2.1; public sector law) and Section 21 of the Act respecting the protection of personal information in the private sector (L.R.Q c. P-39.1; private sector law). Permission to receive the data used for this research was obtained by the *Commission de l’accès à l’information du Québec* and the research protocol received the approval of the University of Montreal Ethics Committee appointed *Comité d’éthique de la recherche en sciences et en santé*.

## Results

### Baseline characteristics

**Figure 1:**
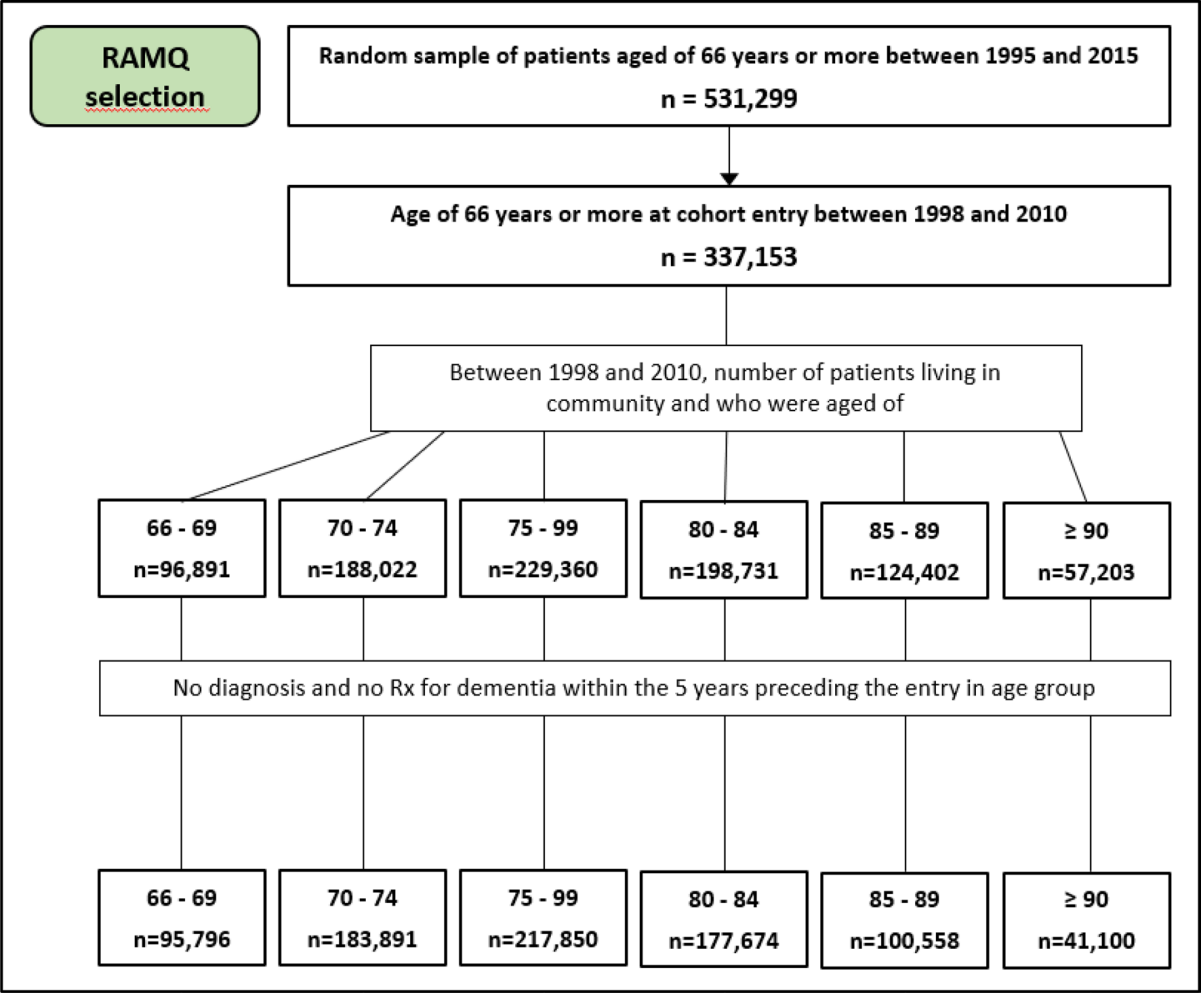
Study flow chart.

The study flow chart is presented at Fig. 1. Demographical and clinical characteristics of the patients are presented in Table 1. The cohort comprises 320,630 patients (42.3% male) with mean age of 74.1 years (standard deviation (SD) = 6.5). The median follow-up time in each age group for the total sample was four years (interquartile range (IQR) 2.0–5.0). During 1,418,752 PY of follow-up, 30,626 patients developed dementia. Incident dementia greatly increased with age, passing from 4.1/1000 PY for patients between 66 and 69 years old to 93.5/1000 PY for those aged ≥ 90. Hypertension, diabetes mellitus and dyslipidemia were the most prevalent CV risk factors, ranging from 14.8 to 61.4% in the different age groups. The prevalence of all CV risk factors increased with age, except for diabetes mellitus and dyslipidemia for which the prevalence was at its highest in patients aged 85–80 and then decrease in patients aged ≥ 90.

**Table 1:** Demographic and clinical characteristics of patients included in the population-based cohorts.

	66–69 (n = 95,796)	70–74 (n = 183,891)	75–79 (n = 217,850)	80–84 (n = 177,674)	85–89 (n = 100,558)	≥ 90 (n = 41,100)
Follow-up time, median years (IQR)	3.0 (1.5–4.0)	4.9 (3.0–5.0)	4.2 (2.2–5.0)	4.0 (2.0–5.0)	3.0 (1.5–5.0)	2.6 (1.1–4.6)
Age at group entry, mean years (SD)	67.4 (1.1)	71.0 (1.4)	75.6 (1.1)	80.4 (1.0)	85.3 (0.9)	90.4 (1.4)
Male (%)	46.7	45.4	43.2	40.0	35.0	28.6
Incident dementia (per 1000 PY)	4.1	9.1	19.9	38.8	63.1	93.5
Mortality rate (per 1000 PY)	15.6	21.3	32.0	49.6	80.2	159.6
Comorbidities in the 5 years prior cohort entry (%)						
Hypertension	40.1	45.7	52.7	57.6	60.9	61.4
Diabetes mellitus	14.8	17.1	19.5	20.4	19.2	15.7
Dyslipidemia	17.8	18.6	19.7	19.9	18.3	14.8
Stroke	2.9	3.6	4.6	5.5	6.3	6.5
Myocardial infarction	2.8	3.1	3.6	4.3	5.2	5.8
Heart failure	3.0	4.1	5.8	8.2	11.5	15.3
Atrial fibrillation	3.5	5.0	7.6	10.9	14.2	16.2
Major bleeding	2.9	3.2	3.7	4.3	4.8	5.2
Systemic embolism	0.5	0.6	0.7	0.7	0.7	0.7
Peripheral artery disease	4.8	6.0	7.7	9.1	10.0	10.1
Kidney disease	3.9	5.0	7.2	10.0	13.0	15.5
Medical procedures in the last 3-year prior to the entry of age group (%)						
Percutaneous coronary intervention—stent/CAGB	2.3	2.6	2.9	2.7	2.3	1.4
Medical procedures for a defibrillator	0.3	0.5	0.7	1.0	1.3	1.4

Medication in 1-year prior to the entry of age group (%)	n = 95,790	n = 182,889	n = 215,393	n = 173,870	n = 97,071	n = 36,376
Diuretics	18.0	21.7	26.1	31.3	37.0	43.7
Loop diuretics	4.2	5.8	8.3	12.1	17.4	24.3
Metolazone	0.1	0.1	0.1	0.1	0.2	0.2
ACEIs/angiotensin receptor blockers	26.4	31.2	36.7	40.5	42.1	41.3
Angiotensin converting enzyme inhibitors	18.4	21.3	24.1	25.9	26.5	25.7
Angiotensin receptor blockers	9.3	11.4	14.2	16.3	17.1	16.8
Beta-blockers	20.2	23.7	27.8	31.7	34.3	34.3
Metoprolol	6.5	7.9	9.6	11.3	12.8	13.3
Carvedilol	0.2	0.2	0.3	0.4	0.4	0.3
Bisoprolol	2.7	3.8	5.8	7.5	8.7	8.8
Other beta-blockers	14.0	16.1	18.6	20.8	22.1	21.7
Spironolactone or eplerenone	0.9	1.1	1.5	1.9	2.4	2.6
Digoxin	1.7	2.2	3.0	4.1	5.5	7.4
Hydralazine	0.1	0.2	0.2	0.3	0.4	0.5
Nitrates	8.0	9.8	11.7	14.0	16.8	20.0
Statins	34.6	39.6	45.0	46.0	41.4	31.1
Antiarrhythmic (amiodarone or propafenone)	0.8	1.1	1.5	1.8	2.0	1.9
Warfarin	3.6	4.9	7.0	9.7	11.9	12.7
Direct oral anticoagulants	0.0	0.1	0.7	1.3	1.9	2.0
Antiplatelets (without ASA)	2.3	3.2	4.4	5.7	6.8	7.7
Low-dose ASA	25.5	31.0	37.3	41.3	43.2	43.4
Antidiabetics						
Metformin	9.4	11.1	13.0	13.2	11.5	8.5
Sulfonylurea	6.3	7.2	7.8	7.8	7.3	6.0
Thiazolidinediones	1.4	1.6	1.5	1.2	0.8	0.5
DPP-4 inhibitors	< 0.1	0.2	0.6	0.8	0.8	0.5
Insulins	2.0	2.3	2.7	2.7	2.4	1.8
Proton pump inhibitors	15.0	18.9	25.2	30.1	33.2	35.0
Antidepressants agents	11.2	12.2	13.5	14.6	15.6	15.5
Anticholinergics agents	2.0	2.1	2.2	2.2	2.3	2.5
Benzodiazepine	23.6	26.4	29.3	32.7	35.6	37.9
Health care services in 1-year prior to the entry of age group						
Number of visit medicals, mean (SD)	6.3 (7.7)	6.6 (8.4)	7.0 (8.9)	6.9 (9.2)	6.6 (9.1)	5.7 (8.1)
Emergency visit, mean (SD)	0.5 (1.3)	0.5 (1.3)	0.6 (1.5)	0.7 (1.6)	0.8 (1.7)	0.9 (1.8)
Hospitalization (%)	13.9	16.0	19.6	22.4	23.3	22.7

*ASA* acetylsalicylic acid, *IQR* interquartile range, *PY* person-years, *SD* standard deviation, *CABG* coronary artery bypass grafting, *ACEIs* angiotensin-converting enzyme inhibitors, *DPP-4* dipeptidyl peptidase 4.

### Incidence rate ratios of dementia among CV risk factors or diseases across age groups

**Figure 2:**
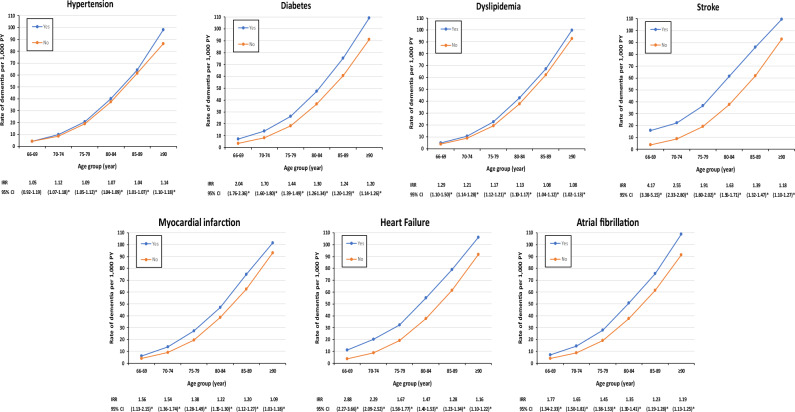
Incidence rate ratios (IRR) of dementia per age group according to cardiovascular risk factors or disease per age groups. *PY* person-years, *IRR* incident rate ratio, *CI* confidence interval, *p < 0.05.

The incident rate ratios (IRR) of dementia for each CV risk factor or disease as a function of age is shown in Fig. 2. IRR of dementia was significant for all CV risk factors or diseases in all age groups except for hypertension in the age group of 66–69 years of age. IRR of dementia for diabetes, stroke, MI, HF and AF ranged from 1.56 to 4.17 in the age group of 66–69 years of age, and then decreased in advanced age.

### Dementia risk and CV risk factors or diseases across age groups

**Figure 3:**
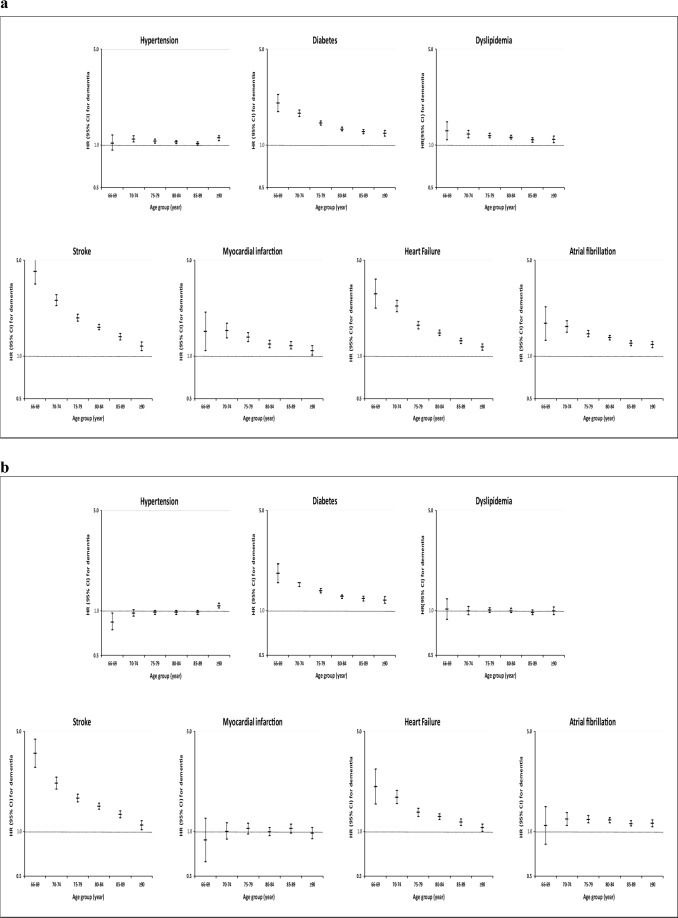
Risk of dementia in the presence of a cardiovascular risk factor or disease across age group. **(a)** Model 1^†^ (^†^Model 1: Cox regression model adjusted for age and sex). **(b)** Model 2^‡^ *HR* hazard ratio, *CI* confidence interval (^‡^Model 2: Cox regression model adjusted for age, sex and all cardiovascular risk factors or diseases).

The HR (95% CI) determined with Cox Regression models (Fig. 3a, Model 1, adjusted for age and sex; Table S2) demonstrated significant associations between dementia and all CV risk factors or diseases, within each age group, except for hypertension in the age group of 66–69 years of age. The analyses performed using competing risks corrected for mortality led to similar results (Fig. S1).

Adjusted HRs for age, sex and all CV risk factors or diseases are reported at Fig. 3b (Model 2) and in Table S2. Hypertension was associated with a decreased risk of dementia from age 66 to 69 years (HR 0.85, 95% CI 0.75–0.98), while it was correlated with an increased dementia risk at age ≥ 90 years (HR 1.10, 95% CI 1.06–1.14). Diabetes mellitus was related with an increased risk of dementia in all group ages, with HR decreasing from 1.84 (95% CI 1.84–2.13) at age 66–69 years to 1.20 (95% CI 1.14–1.26) at age ≥ 90 years. Dyslipidemia was not linked with an increase of dementia risk. Stroke was associated with an increased risk of dementia in all group ages but the dementia risk decreased with advanced age (HR of 3.54 (95% CI 2.83–4.42) at age 66–69 years to 1.13 (95% CI 1.05–1.21) at age ≥ 90 years). MI was not linked with an increased dementia risk. HF was correlated with an increased dementia risk in all group ages. The HR decreased from 2.08 (95% CI 1.57–2.74) at age 66–69 years to 1.16 (95% CI 1.02–1.14) at age ≥ 90 years. On its end, AF was linked with an increased risk of dementia in age ≥ 70 years and over, but the association was more or less stable with advanced ages (HR of 1.24 (95% CI 1.12–1.37) at age 70–74 years to 1.16 (95% CI 1.10–1.22) at age ≥ 90 years). Adjusting for additional risk factors or diseases yielded similar results (Table S3).

### Sensitivity analysis

We provided sensitivity analyses among women and men across age-groups. The IRR of dementia for each CV risk factor or disease as a function of age-group and sex are shown in Fig. S2a and Fig. S2b. And, as presented in Table S4, the risk factors exhibit similar results among women and men across age-groups after adjustment for other CV risk factors or diseases, except age-group 66 to 69 years old. For instance, in the age-group 66–69, dyslipidemia was linked with an increase of dementia risk only in women; And, chronic heart failure, major bleeding, chronic kidney disease seemed to be an increasing risk factor of dementia mainly in women compared to men.

## Discussion

After adjustment for other CV risk factors or diseases, diabetes, stroke, HF and AF remained significantly associated with an increased dementia risk in all aged groups studied. The strength of these associations decreased with advanced age for diabetes, stroke and HF. From 66 to over 90 years, the increased dementia risk associated with respectively diabetes, stroke and HF passed from 1.84, 3.54, 2.08 folds in the younger age group to 1.20, 1.13, 1.16 among patients ≥ 90 years old. The association of AF and dementia was more or less stable with advanced ages. Our results suggest that preventing diabetes, stroke, HF and AF before early advanced age is crucial to prevent dementia. So, cognitive function assessment should be performed in the routine clinical management of patients with CV risk factors, especially in early ages, and a decline in cognitive function should militate for a more aggressive control of CV risk factors^31^. It also suggests that pathophysiology of dementia in younger and older populations seems to be different with less impact of CV risk factors or disease in advanced age.

The age-dependent effect of dementia in older populations has been described previously^19,32^. Recently, Legdeur et al*.* found a similar pattern of decrease in the risk of CV risk factors or diseases studies for incident dementia with aging in patients ≥ 65 years, but only up to 90 years^19^. And, our results are also well aligned with the study of McGrath et al*.*, where vascular risk factors and dementia risk prediction across later-life from Framingham Heart Study for hypertension, diabetes, AF and stroke. For instance, the age group 75–80, the HRs of hypertension, diabetes, AF and stroke were at 0.74, 1.40, 1.43 and 1.63 compared to our results 0.99, 1.39,1.23, 1.73, respectively^32^. Previous studies had also reported age-dependant effects on incident dementia risk for hypertension^20,33–36^, dyslipidemia^20,37^, AF, MI^38^, and a risk score that included diabetes mellitus, hypertension, dyslipidemia and MI^39^. They found that these risk factors are associated with dementia in age 40–65 years, but not in age > 65 years, excepted for the risk score that was associated with dementia up to the age of 79 years. For diabetes mellitus, it is known that diabetes increases dementia risk, that the risk is stronger when diabetes occurs at mid-life than in late life^40^; and that this association remains in patients ≥ 85 years^20,41–43^. For stroke as well, we noted that the dementia risk decreased with aging, and the association of stroke with incident dementia persisted in patients ≥ 85 years^33^. It should be noted that study design used, adjustments for CV risk factors or diseases, medication exposure, and the type of outcome (all cause dementia, versus vascular dementia or Alzheimer’s disease) differed between studies, prohibiting an adequate direct comparison of effect estimates over studies.

Many specific risk-factors have been put forward to explain the observed age-dependant effect. First, it is suggested that atherosclerosis resulting from high blood pressure, and cerebral hypoperfusion related to severe atherosclerosis and to low blood pressure could explain the pathways linking high blood pressure in younger patients and low blood pressure in older adults to cognitive decline and dementia^33,35^. Additionally, this could also be explained by the complex perspective of age-associated changes in cardiovascular structure and its cross talk with blood pressure variability. Thickening and stiffening of the large arteries develop with aging due to collagen and calcium deposition and loss of elastic fibers in the medial layer^44^. In the left ventricle, modest concentric wall thickening occurs with ageing due to cellular hypertrophy^44^. These arterial changes cause systolic blood pressure to rise with age, while diastolic blood pressure generally declines, resulting in an increased pulse pressure variability. Simultaneous multi-organ dysfunction (defined as simultaneous occurrence of left ventricular hypertrophy, common carotid artery disease and increased thickness, and chronic kidney disease) has been associated with low diastolic blood pressure rather than systolic hypertension^45^. So, the decrease in diastolic blood pressure with age might explain more damageable than systolic hypertension, explaining the decrease in the association between hypertension and dementia age^45^. However, in our study, hypertension and dyslipidemia are not associated with dementia risk, when adjusted for the other CV risk factors or diseases. Second, higher cholesterol values was associated with a decreased dementia risk in late life, and may indirectly indicate a better overall health status in older patients^46^. Third, there might be a reverse causality for the age-dependant effect of hypertension, dyslipidemia and diabetes with blood pressure, cholesterol and glucose levels may drop before the onset of the neurodegenerative process^35,47^.

Hypothesis generalizable to all risk factors may explain the observed age-dependent effect as well. First, there may be a selective survival of patients who are less likely to develop dementia as consequence of the risk factors. Second, the higher prevalence of CV risk factors and other non-CV risk factors of dementia in elderly patients attenuates the distinction of patients with and without a CV risk factors^48^. Nonetheless, a similar decrease in the dementia risk was observed when additional adjustment for any of the other CV risk factors or diseases included in our study was performed, but other CV and non-CV risk factors may confound our observations. Third, patients with CV risk factors may die earlier than those without the risk factor, and are consequently less likely to develop dementia before their death. Notwithstanding, adjusting for mortality as a competing risk led to similar results in our study, with the risk of CV risk factors or diseases for incident dementia diminishes with increasing age (except for hypertension, dyslipidemia and MI). Fourth, neuropathological studies have shown that the vascular risk burden was predictive of vascular dementia (cortical infarcts, subcortical infarcts, atherosclerosis, and arteriosclerosis), but not Alzheimer’s disease (Braak stage, cerebral amyloid angiopathy and, neuritic plaque score)^49^. However, neuropathological studies have also revealed that, in late life, the presence of multiples pathologies (Alzheimer’s disease, microinfarcts, hippocampal sclerosis, Lewy body disease, macroinfarcts, cerebral amyloid angiopathy, white matter disease, and others) were associated with an increased risk of dementia^50^. Accordingly, the observed decreasing risk of CV risk factors or diseases for incident dementia with increasing age might be due to the fact that our studied outcome was all-cause dementia and that the presence of multiple pathologies, rather than vascular dementia on its own, seems to be involved in the development as age increase. Fifth, we should have been taking in account the CV risk level in the risk prediction of dementia with longitudinal data since, the improvement of CV modifiable risk was associated with a reduction of dementia risk^51–53^. Finally, we did not take in account the impact of level adherence of CV medication.

Our study is one of the largest to examine the effect of age on risk of CV risk factors or diseases on the incidence of dementia in a population ranging from 66 to over 90 years. The strengths and limitations of our study are greatly related to the nature of the Quebec RAMQ administrative databases. As there is single payer public health care system in Quebec (Canada), Quebec RAMQ administrative database includes all individuals over 65 years old, limiting selection bias, and captures all diagnosis, procedures and drugs. However, several limitations must be taken into consideration. First, administrative claims data depend on the exhaustive, accurate recording and coding of diagnoses, procedures, and drugs. Second, this observational study of administrative data might have been subject to confounding bias by unadjusted factors (e.g., auto-immune diseases, pro-inflammatory conditions, race, level of education, smoking, sedentary lifestyle, major depressive disorder, etc.). Third, administrative claims data depend on the exhaustive, accurate recording and coding of diagnoses and procedures. Fourth, the accurate starting for CV risk factors or diseases may not always accurately recorded in administrative claims data, so the duration of the CV disorders could not be accounted for, but we have used a 5-year period to assess risk CV risk factors or diseases. Fifth, no distinction between types of dementia was performed, as the type of dementia is not reliably specified in administrative claims data. Sixth, quantitative measurements of blood pressure, glucose and cholesterol were not available and we could not take in account appropriate control of hypertension, diabetes mellitus and cholesterol. Seventh, a patient may be included in more than one age group during the follow-up in order to consider the cohort risk according to each age-group, which may introduce a survival bias. We thus provided a sensitivity analysis using competing risks correcting for mortality, and this led to similar results. Again, the risk factors of dementia exhibit similar results by stratifying by sex across age-groups. Last, we could not adjust for factors such as, economic, health status, severity of disease, lifestyle and education that could be also linked with dementia risk^54^.

## Conclusion

We found the strength of association between CV risk factors or diseases on the incidence of dementia change with age, except for hypertension, dyslipidemia, and MI. Diabetes, stroke, HF and AF were the main risks factors for dementia, and their burden for dementia diminishes with increasing age from 66 to over 90 years, except for AF. Future research and clinical initiatives should pay special attention to age-related impact of different CV risk factors or diseases on the incidence of dementia. Our results also suggest that dementia may be explained by different pathophysiological processes in young-old vs. old-old adults.

### Supplementary Information

Supplementary Information.

## Data Availability

All data generated or analysed during this study are included in this published article and its supplementary information file.
