# Supplementary material for: Association between cardiovascular diseases and dementia among various age groups: a population-based cohort study in older adults
Source: Sci Rep. 2023 Sep 9;13:14881. doi: 10.1038/s41598-023-42071-8 (PMC10492794; doi:10.1038/s41598-023-42071-8)
Supplement: Supplementary file 1 — Supplementary Information. [file 41598_2023_42071_MOESM1_ESM.pdf]

Supplementary Information

Association between Cardiovascular Diseases and Dementia among various age groups: A Population-Based Cohort Study in Older Adults

Laurie-Anne Boivin-Proulx[1], Judith Brouillette[1,2], Marc Dorais[3], Sylvie Perreault[4,5*]

[1] Department of Cardiology, Faculty of Medicine, University of Ottawa Heart Institute, Ottawa, Ontario, Canada.

[2] Department of Psychiatry and Addictology, Université de Montréal, Montreal, Quebec, Canada.

[3] StatSciences Inc., Notre-Dame-de-l'Île-Perrot, Quebec, Canada.

[4] Faculty of Pharmacy, Université de Montréal, Montreal, Quebec, Canada.

[5] Centre de recherche en santé publique (CReSP), partenaire CIUSSS du Centre-Sud-de-l'Île-de-Montréal et l'Université de Montréal, Montreal, Quebec, Canada.

***Corresponding Author:**
Sylvie Perreault, BPharm, PhD,
Faculté de Pharmacie, Université de Montréal, Case Postale 6128, Succursale Centre-Ville, Montréal, Québec, Canada, H3C 3J7
Centre de recherche en santé publique (CReSP), partenaire CIUSSS du Centre-Sud-de-l'Île-de-Montréal et l'Université de Montréal
Tel: +1 (514) 951-3149
E-mail: sylvie.perreault@umontreal.ca

	ICD-9 codes	ICD-10 codes
**Hypertension**	401	I10
**Diabetes**	250.x	E08, E10, E11, E13
**Dyslipidemia**	272	E78
**Stroke**		
Hemorrhagic stroke intracranial (non-traumatic)	430, 431, 432.x	I60, I61, I62
Ischaemic stroke	433.xx, 434.xx, 436.0, 436.9	I63 except I63.6, I64
Transient ischemic attack	435.x	G45
**Myocardial infarction**	410.xx	I21, I22, I23
**Chronic heart failure**	428.0, 428.1, 428.9	I50, I50.1, I50.9
**Atrial fibrillation**	427.3	I48
**Major bleeding**		
**-Intracranial major bleeding**		
Intracranial bleeding	430, 431, 432.x, 852.x, 853.x	I60, I61, I62, S06.3, S06.4, S06.5, S06.6
**-Major gastrointestinal bleeding**		
Upper gastrointestinal bleeding (only using Med-Echo)	456.1, 530.7, 531.0x, 531.2x, 531.4x, 531.6x, 532.0x, 532.2x, 532.4x. 532.6x, 533.0x. 533.2x, 533.4x, 533.6x, 534.0x, 534.2x, 534.4x, 534.6x, 535.1, 578.0	I85.0, K22.6, K25.0, K25.2, K25.4, K25.6, K26.0, K26.2, K26.4, K26.6, K27.0, K27.2, K27.4, K27.6, K28.0, K28.2, K28.4, K28.6, K29.0, K92.0
Upper gastrointestinal bleeding (only using RAMQ)	456.1, 530.7, 531.0x, 531.2x, 531.4x, 531.6x, 532.0x, 532.2x, 532.4x. 532.6x, 533.0x, 533.2x, 533.4x, 533.6x, 534.0x, 534.2x, 534.4x, 534.6x, 535.1, 578.0 RAMQ ICD-9 at an emergency room and procedure endoscopic control of gastric or duodenal bleeding or upper gastrointestinal endoscopy including esophagus, stomach, and either the duodenum and/or jejunum as appropriate with control of bleeding, any method (code 00691) within 7 days	I85.0, K22.6, K25.0, K25.2, K25.4, K25.6, K26.0, K26.2, K26.4, K26.6, K27.0, K27.2, K27.4, K27.6, K28.0, K28.2, K28.4, K28.6, K29.0, K92.0 RAMQ ICD-9 at an emergency room and procedure endoscopic control of gastric or duodenal bleeding or upper gastrointestinal endoscopy including esophagus, stomach, and either the duodenum and/or jejunum as appropriate with control of bleeding, any method (00691) within 7 days

Lower gastrointestinal bleeding	562.02, 562.03, 562.12, 562.13, 569.3x, 569.85, 578.1x, 578.9	K57.11, K57.13, K57.31, K57.33, K62.5, K55.21, K92.1, K92.2
**-Other critical sites of major bleeding**		
Gross hematuria	599.7	R31
Hemoptysis	786.3x	R04.2, R04.89, R04.9
Vitreous hemorrhage	379.23	H43.13
Urogenital bleed	626.2x and 280.0, 285.1 or 285.9	N92.0 and D50.0, D62, D64.9)
Hemarthrosis	719.1x	M25.0x
Hemopericardium	423.0	I31.2
Hemoperitoneum	568.8	K66.1
Hemorrhage not specified	459.0x	R58.0
Acute posthemorrhagic anemia	285.1x	D62
**-Systemic embolism**	444.x, 557.0, 362.31, 362.32, 598.31	I74
Arterial embolism and thrombosis	444.x	I74.0, I74.1, I74.2, I74.3, I74.5, I74.8, I74.9
Ischemic colitis or mesenteric thromboembolism	557.0	K55.0
Retinal artery thromboembolism	362.31, 362.32	H34.1, H34.2
Renal artery thromboembolism	593.81	N28.0
**Peripheral arterial disease**	440 (except 440.0), 441, 443.0, 443.89, 443.9	I70.1 to I70.9, I71, I73.0, I73.89, I73.9
**Chronic kidney disease** (moderate to severe disease)	404.01, 404.03, 404.11, 404.13, 404.91, 404.93, 580.0, 580.4, 581.0, 581.1, 581.2, 581.3, 581.89, 581.9, 582.0, 582.1, 582.2, 582.89, 582.9, 583.0, 583.1, 583.2, 583.4, 583.7, 583.6, 583.89, 583.9, 584.5, 584.6, 584.7, 584.8, 584.9, 585.1, 585.2, 585.3, 585.4, 585.5, 585.6, 586, 590.0, 590.01, 590.80	I12, I13, N00, N01, N02, N03, N04, N05, N07, N11, N12, N14, N17, N18, N19

Table S1. Definition of cardiovascular risk factors or diseases according to ICD-9 and ICD-10 from Med-Echo databases or RAMQ medical service files.

ICD-9 International Classification of Diseases - 9th Revision, *ICD-10* International Classification of Diseases - 10th Revision, *RAMQ* Régie de l'assurance maladie du Québec, *Med-Echo* Administrative databases of hospital discharge reports.

	Age group 66–69		Age group 70–74	
	HR (95% CI)		HR (95% CI)	
**Risk factors**	Model 1 Adjusted for age and sex†	Model 2 Multivariate‡	Model 1 Adjusted for age and sex †	Model 2 Multivariate‡
Hypertension	1.05 (0.93–1.20)	0.85 (0.75–0.98)	1.12 (1.07–1.18)	0.98 (0.93–1.03)
Diabetes mellitus	2.03 (1.76–2.35)	1.84 (1.58–2.13)	1.71 (1.62–1.81)	1.58 (1.49–1.68)
Dyslipidemia	1.28 (1.10–1.49)	1.04 (0.88–1.22)	1.21 (1.14–1.29)	1.01 (0.95–1.08)
Stroke	4.16 (3.37–5.15)	3.54 (2.83–4.42)	2.57 (2.35–2.82)	2.19 (1.99–2.41)
Myocardial infarction	1.53 (1.11–2.11)	0.89 (0.63–1.26)	1.55 (1.37–1.75)	1.02 (0.90–1.17)
Chronic heart failure	2.87 (2.26–3.65)	2.08 (1.57–2.74)	2.33 (2.12–2.56)	1.76 (1.58–1.96)
Atrial fibrillation	1.75 (1.32–2.30)	1.12 (0.83–1.51)	1.66 (1.51–1.83)	1.24 (1.12–1.37)
	Age group 75–79		Age group 80–84	
	HR (95% CI)		HR (95% CI)	
**Risk factors**	Model 1 Adjusted for age and sex †	Model 2 Multivariate‡	Model 1 Adjusted for age and sex †	Model 2 Multivariate‡
Hypertension	1.08 (1.05–1.12)	0.99 (0.96–1.02)	1.07 (1.04–1.09)	0.99 (0.96–1.02)
Diabetes mellitus	1.46 (1.41–1.51)	1.39 (1.34–1.44)	1.32 (1.28–1.36)	1.27 (1.23–1.30)
Dyslipidemia	1.18 (1.14–1.23)	1.02 (0.98–1.06)	1.15 (1.12–1.19)	1.01 (0.98–1.05)
Stroke	1.92 (1.81–2.03)	1.73 (1.63–1.84)	1.64 (1.57–1.72)	1.52 (1.45–1.60)
Myocardial infarction	1.39 (1.29–1.50)	1.07 (0.98–1.16)	1.24 (1.17–1.32)	1.01 (0.95–1.08)
Chronic heart failure	1.70 (1.60–1.80)	1.38 (1.29–1.47)	1.49 (1.43–1.56)	1.29 (1.23–1.35)
Atrial fibrillation	1.47 (1.40–1.55)	1.23 (1.17–1.31)	1.38 (1.33–1.43)	1.22 (1.17–1.27)
	Age group 85–89		Age group ≥ 90	
	HR (95% CI)		HR (95% CI)	
**Risk factors**	Model 1 Adjusted for age and sex †	Model 2 Multivariate‡	Model 1 Adjusted for age and sex†	Model 2 Multivariate‡
Hypertension	1.04 (1.01–1.07)	0.99 (0.96–1.02)	1.14 (1.09–1.18)	1.10 (1.06–1.14)
Diabetes mellitus	1.26 (1.22–1.31)	1.23 (1.18–1.27)	1.23 (1.17–1.29)	1.20 (1.14–1.26)
Dyslipidemia	1.10 (1.06–1.14)	0.98 (0.95–1.02)	1.11 (1.05–1.17)	1.01 (0.95–1.07)
Stroke	1.40 (1.33–1.48)	1.34 (1.27–1.41)	1.20 (1.11–1.28)	1.13 (1.05–1.21)
Myocardial infarction	1.21 (1.14–1.29)	1.07 (0.99–1.14)	1.11 (1.03–1.21)	0.99 (0.91–1.08)
Chronic heart failure	1.31 (1.25–1.36)	1.18 (1.12–1.24)	1.18 (1.12–1.24)	1.08 (1.02–1.14)
Atrial fibrillation	1.26 (1.21–1.31)	1.15 (1.11–1.21)	1.23 (1.17–1.29)	1.16 (1.10–1.22)

Table S2. Association between the cardiovascular risk factors or diseases and dementia risk with Cox regression models.

HR hazard ratio, *CI* confidence interval.

† Model 1: Cox regression model adjusted for age and sex.

‡ Model 2: Cox regression model adjusted for age, sex and all cardiovascular risk factors or diseases.

	Age group 66–69		Age group 70–74	
	HR (95% CI)		HR (95% CI)	
**Risk factors**	Model 1† Adjusted for age and sex	Model 2‡ Multivariate	Model 1† Adjusted for age and sex	Model 2‡ Multivariate
Hypertension	1.05 (0.93–1.20)	0.84 (0.73–0.96)	1.12 (1.07–1.18)	0.96 (0.91–1.01)
Diabetes mellitus	2.03 (1.76–2.35)	1.73 (1.48–2.03)	1.71 (1.62–1.81)	1.48 (1.40–1.57)
Dyslipidemia	1.28 (1.10–1.49)	1.02 (0.86–1.20)	1.21 (1.14–1.29)	0.96 (0.90–1.03)
Stroke	4.16 (3.37–5.15)	3.33 (2.63–4.20)	2.57 (2.35–2.82)	1.92 (1.74–2.13)
Myocardial infarction	1.53 (1.11–2.11)	0.87 (0.61–1.23)	1.55 (1.37–1.75)	0.95 (0.83–1.09)
Chronic heart failure	2.87 (2.26–3.65)	1.88 (1.42–2.51)	2.33 (2.12–2.56)	1.55 (1.39–1.73)
Atrial fibrillation	1.75 (1.32–2.30)	1.09 (0.80–1.46)	1.66 (1.51–1.83)	1.19 (1.08–1.32)
Major bleeding	1.97 (1.49–2.62)	1.48 (1.11–1.97)	1.67 (1.49–1.87)	1.34 (1.19–1.51)
Systemic embolism	1.98 (1.03–3.82)	1.01 (0.51–2.00)	2.13 (1.68–2.71)	1.08 (0.85–1.39)
Peripheral artery disease	1.84 (1.46–2.33)	1.01 (0.77–1.33)	2.03 (1.87–2.20)	1.43 (1.30–1.57)
Chronic kidney disease	2.56 (2.06–3.24)	1.46 (1.13–1.89)	2.25 (2.07–2.45)	1.47 (1.34–1.62)
	Age group 75–79		Age group 80–84	
	HR (95% CI)		HR (95% CI)	
**Risk factors**	Model 1† Adjusted for age and sex	Model 2‡ Multivariate	Model 1† Adjusted for age and sex	Model 2‡ Multivariate‡
Hypertension	1.08 (1.05–1.12)	0.97 (0.94–1.00)	1.07 (1.04–1.09)	0.98 (0.95–1.00)
Diabetes mellitus	1.46 (1.41–1.51)	1.33 (1.28–1.38)	1.32 (1.28–1.36)	1.24 (1.20–1.27)
Dyslipidemia	1.18 (1.14–1.23)	0.98 (0.94–1.02)	1.15 (1.12–1.19)	0.98 (0.95–1.02)
Stroke	1.92 (1.81–2.03)	1.61 (1.52–1.71)	1.64 (1.57–1.72)	1.47 (1.40–1.54)
Myocardial infarction	1.39 (1.29–1.50)	1.01 (0.94–1.10)	1.24 (1.17–1.32)	0.98 (0.92–1.05)
Chronic heart failure	1.70 (1.60–1.80)	1.27 (1.19–1.36)	1.49 (1.43–1.56)	1.23 (1.17–1.29)
Atrial fibrillation	1.47 (1.40–1.55)	1.20 (1.13–1.27)	1.38 (1.33–1.43)	1.20 (1.15–1.25)
Major bleeding	1.52 (1.41–1.63)	1.31 (1.22–1.41)	1.28 (1.20–1.35)	1.13 (1.06–1.20)
Systemic embolism	1.51 (1.28–1.78)	0.99 (0.84–1.18)	1.16 (0.99–1.35)	0.88 (0.75–1.03)
Peripheral artery disease	1.56 (1.49–1.65)	1.23 (1.16–1.30)	1.35 (1.29–1.40)	1.15 (1.10–1.20)
Chronic kidney disease	1.71 (1.63–1.80)	1.31 (1.24–1.39)	1.43 (1.37–1.48)	1.18 (1.13–1.23)
	Age group 85–89		Age group ≥ 90	
	HR (95% CI)		HR (95% CI)	
**Risk factors**	Model 1† Adjusted for age and sex	Model 2‡ Multivariate	Model 1† Adjusted for age and sex	Model 2‡ Multivariate
Hypertension	1.04 (1.01–1.07)	0.98 (0.95–1.01)	1.14 (1.09–1.18)	1.08 (1.04–1.13)
Diabetes mellitus	1.26 (1.22–1.31)	1.20 (1.16–1.25)	1.23 (1.17–1.29)	1.18 (1.12–1.24)
Dyslipidemia	1.10 (1.06–1.14)	0.95 (0.91–0.99)	1.11 (1.05–1.17)	0.98 (0.92–1.03)
Stroke	1.40 (1.33–1.48)	1.30 (1.23–1.37)	1.20 (1.11–1.28)	1.10 (1.03–1.19)
Myocardial infarction	1.21 (1.14–1.29)	1.03 (0.97–1.10)	1.11 (1.03–1.21)	0.97 (0.89–1.05)
Chronic heart failure	1.31 (1.25–1.36)	1.13 (1.08–1.19)	1.18 (1.12–1.24)	1.04 (0.98–1.10)
Atrial fibrillation	1.26 (1.21–1.31)	1.13 (1.09–1.18)	1.23 (1.17–1.29)	1.14 (1.08–1.20)
Major bleeding	1.22 (1.15–1.30)	1.11 (1.04–1.19)	1.10 (1.02–1.19)	1.02 (0.94–1.10)
Systemic embolism	1.36 (1.17–1.59)	1.11 (0.95–1.30)	1.02 (0.81–1.29)	0.84 (0.67–1.07)
Peripheral artery disease	1.27 (1.21–1.33)	1.13 (1.08–1.19)	1.23 (1.16–1.31)	1.14 (1.07–1.21)
Chronic kidney disease	1.33 (1.27–1.38)	1.17 (1.12–1.22)	1.29 (1.22–1.36)	1.17 (1.11–1.24)

Table S3. Association between the cardiovascular risk factors or diseases and dementia risk with Cox regression models and including major bleeding, systemic embolism, peripheral artery disease and chronic kidney disease.

HR hazard ratio, *CI* confidence interval.
† Model 1: Cox regression model adjusted for age and sex.
‡ Model 2: Cox regression model adjusted for age, sex and all cardiovascular risk factors or diseases and including major bleeding, systemic embolism, peripheral artery disease and chronic kidney disease.

	Age group 66–69		Age group 70–74	
	HR (95% CI)		HR (95% CI)	
**Risk factors**	Women<br>Model 2‡<br>Multivariate	Men<br>Model 2‡<br>Multivariate	Women<br>Model 2‡<br>Multivariate	Men<br>Model 2‡<br>Multivariate
Hypertension	**0.89 (0.77–1.08)**	0.79 (0.65–0.96)	0.99 (0.92–1.06)	0.93 (0.86–1.00)
Diabetes mellitus	1.51 (1.19–1.91)	1.90 (1.54–2.34)	1.49 (1.37–1.62)	1.46 (1.34–1.60)
Dyslipidemia	**1.27 (1.01–1.59)**	0.81 (0.61–1.03)	1.01 (0.93–1.11)	0.91 (0.83–1.00)
Stroke	3.32 (2.36–4.69)	3.35 (2.44–4.61)	1.81 (1.56–2.10)	2.03 (1.77–2.32)
Myocardial infarction	1.01 (0.60–1.73)	0.83 (0.52–1.32)	0.99 (0.80–1.23)	0.94 (0.79–1.12)
Chronic heart failure	2.49 (1.68–3.91)	**1.46 (0.97–2.21)**	1.40 (1.19–1.65)	1.69 (1.45–1.95)
Atrial fibrillation	1.04 (0.67–1.63)	1.14 (0.76–1.71)	1.37 (1.18–1.59)	**1.07 (0.97–1.23)**
Major bleeding	1.77 (1.19–1.63)	**1.24 (0.82–1.89)**	1.33 (1.13–1.58)	1.34 (1.14–1.58)
Systemic embolism	Sample size too small	1.59 (0.76–3.33)	1.00 (0.68–1.46)	1.14 (0.83–1.58)
Peripheral artery disease	0.86 (0.55–1.34)	1.16 (0.83–1.63)	1.58 (1.38–1.82)	1.33 (1.18–1.51)
Chronic kidney disease	1.60 (1.10–2.33)	**1.38 (0.97–1.95)**	1.58 (1.38–1.81)	1.39 (1.22–1.58)
	Age group 75–79		Age group 80–84	
	HR (95% CI)		HR (95% CI)	
**Risk factors**	Women<br>Model 2‡<br>Multivariate	Men<br>Model 2‡<br>Multivariate	Women<br>Model 2‡<br>Multivariate	Men<br>Model 2‡<br>Multivariate
Hypertension	0.98 (0.94–1.02)	0.96 (0.91–1.01)	0.97 (0.94–1.01)	0.99 (0.94–1.03)
Diabetes mellitus	1.36 (1.29–1.43)	1.29 (1.22–1.37)	1.23 (1.18–1.28)	1.24 (1.18–1.30)
Dyslipidemia	1.03 (0.97–1.08)	**0.92 (0.87–0.98)**	0.99 (0.94–1.03)	0.98 (0.93–1.03)
Stroke	1.61 (1.48–1.76)	1.61 (1.48–1.76)	1.43 (1.33–1.53)	1.51 (1.41–1.63)
Myocardial infarction	1.04 (0.92–1.17)	1.00 (0.90–1.11)	1.06 (0.97–1.16)	0.91 (0.83–1.00)
Chronic heart failure	1.23 (1.12–1.35)	1.31 (1.20–1.44)	1.21 (1.14–1.30)	1.25 (1.16–1.34)
Atrial fibrillation	1.24 (1.14–1.34)	1.17 (1.08–1.27)	1.26 (1.19–1.33)	1.13 (1.06–1.20)
Major bleeding	1.32 (1.20–1.46)	1.29 (1.16–1.43)	1.15 (1.06–1.25)	1.10 (1.00–1.20)
Systemic embolism	1.00 (0.78–1.28)	0.99 (0.78–1.24)	0.86 (0.69–1.07)	0.90 (0.72–1.12)
Peripheral artery disease	1.22 (1.12–1.33)	1.25 (1.16–1.35)	1.14 (1.06–1.21)	1.15 (1.08–1.23)
Chronic kidney disease	1.34 (1.24–1.46)	1.29 (1.19–1.39)	1.21 (1.14–1.28)	1.17 (1.14–1.21)
	Age group 85–89		Age group ≥ 90	
	HR (95% CI)		HR (95% CI)	
**Risk factors**	Women<br>Model 2‡<br>Multivariate	Men<br>Model 2‡<br>Multivariate	Women<br>Model 2‡<br>Multivariate	Men<br>Model 2‡<br>Multivariate
Hypertension	0.97 (0.93–1.00)	0.99 (0.94–1.04)	1.09 (1.05–1.14)	**1.06 (0.98–1.14)**
Diabetes mellitus	1.19 (1.13–1.24)	1.23 (1.16–1.31)	1.16 (1.09–1.22)	1.24 (1.12–1.36)
Dyslipidemia	**0.96 (0.91–1.01)**	0.93 (0.87–0.99)	1.00 (0.93–1.06)	0.92 (0.83–1.03)
Stroke	1.22 (1.13–1.31)	1.43 (1.31–1.55)	**1.06 (0.97–1.16)**	1.22 (1.07–1.40)
Myocardial infarction	1.03 (0.94–1.12)	1.04 (0.94–1.15)	0.98 (0.88–1.08)	0.95 (0.82–1.11)
Chronic heart failure	1.14 (1.07–1.21)	1.11 (1.03–1.21)	1.03 (0.97–1.11)	1.07 (0.96–1.20)
Atrial fibrillation	1.16 (1.10–1.22)	1.10 (1.03–1.18)	1.15 (1.08–1.22)	1.13 (1.02–1.25)
Major bleeding	1.11 (1.02–1.20)	1.12 (1.01–1.24)	0.97 (0.88–1.07)	1.14 (0.99–1.31)
Systemic embolism	1.14 (0.94–1.39)	1.04 (0.80–1.36)	0.86 (0.66–1.13)	0.80 (0.50–1.27)
Peripheral artery disease	1.15 (1.08–1.23)	1.10 (1.02–1.19)	1.16 (1.07–1.25)	**1.11 (0.99–1.24)**
Chronic kidney disease	1.17 (1.11–1.24)	1.16 (1.08–1.25)	1.18 (1.10–1.26)	1.16 (1.05–1.28)

Table S4. Association between the cardiovascular risk factors or diseases and dementia risk for women and men with Cox regression models and including major bleeding, systemic embolism, peripheral artery disease and chronic kidney disease.

HR hazard ratio, *CI* confidence interval.
[†] Model 1: Cox regression model adjusted for age
[‡] Model 2: Cox regression model adjusted for age and all cardiovascular risk factors or diseases and including major bleeding, systemic embolism, peripheral artery disease and chronic kidney disease.

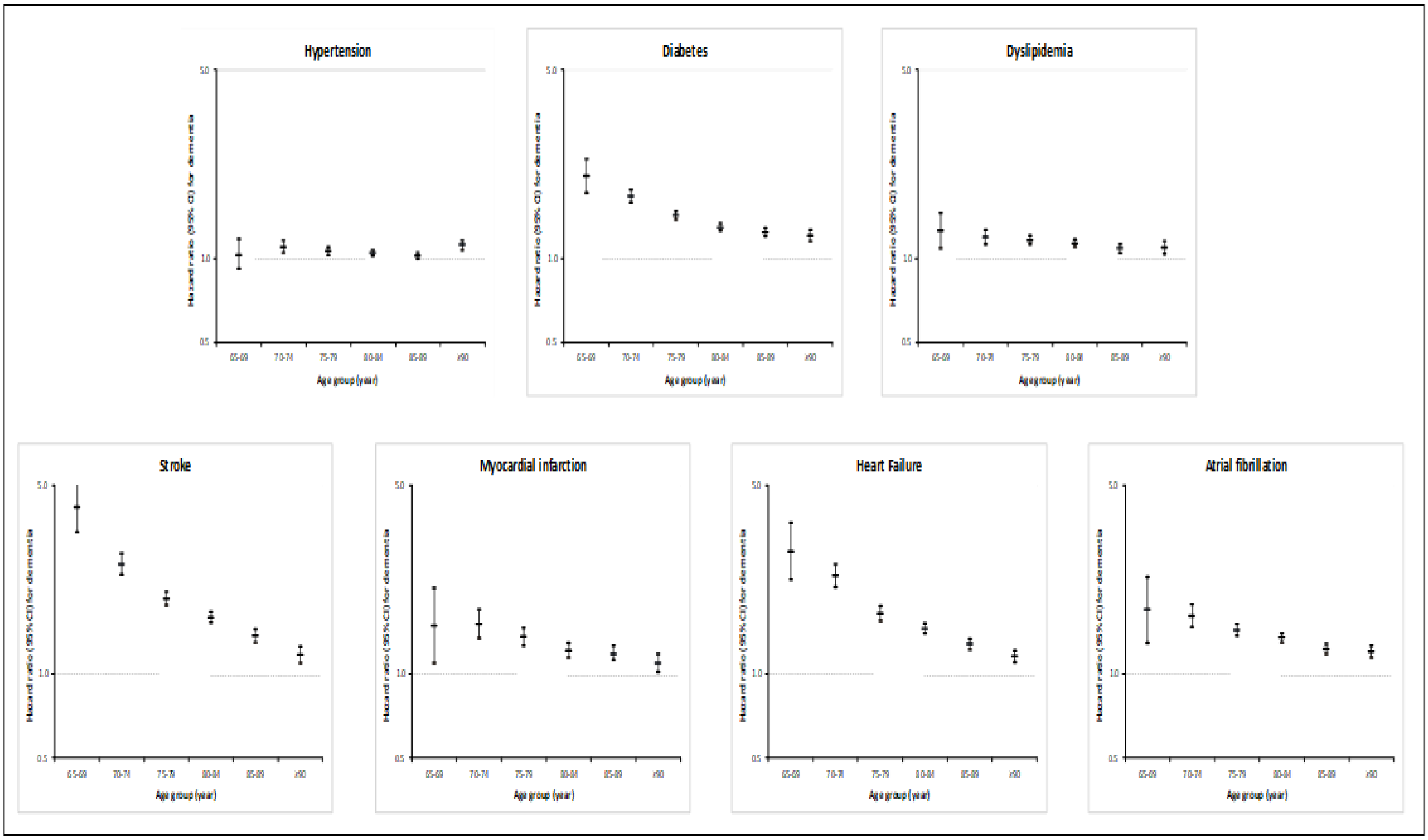

Figure S1. Risk of dementia in the presence of cardiovascular risk factors or diseases per 5-year age group (except for patients ≥ 90, in which no upper age limit was used) determined with competing risks, adjusted for age at cohort entry and sex.

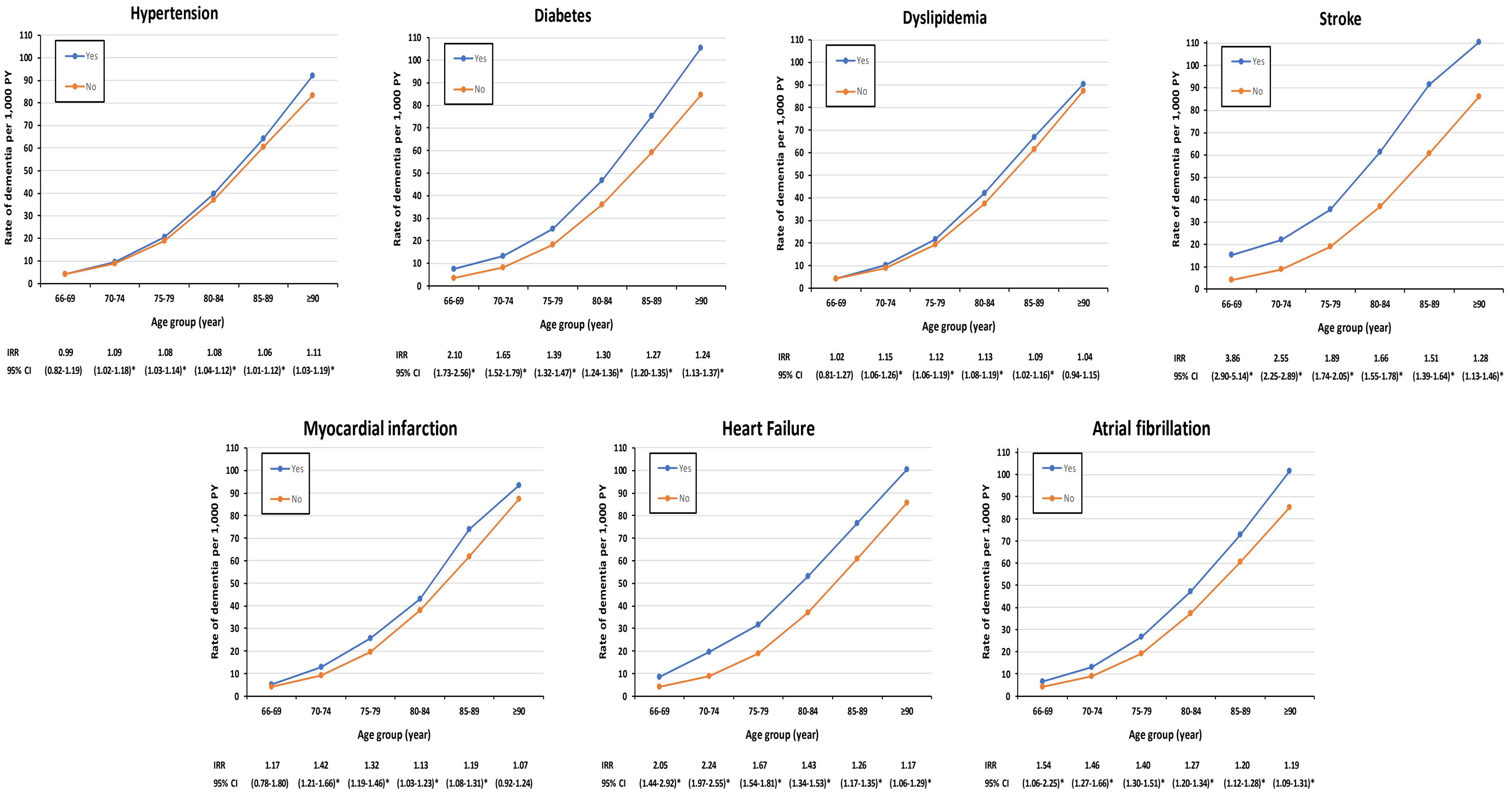

Figure S2a. Incidence Rate Ratios (IRR) of dementia in women per age group according to cardiovascular risk factors or disease per age groups. *PY* person-years, *IRR* incident rate ratio, *CI* confidence interval, * $p < 0.05$.

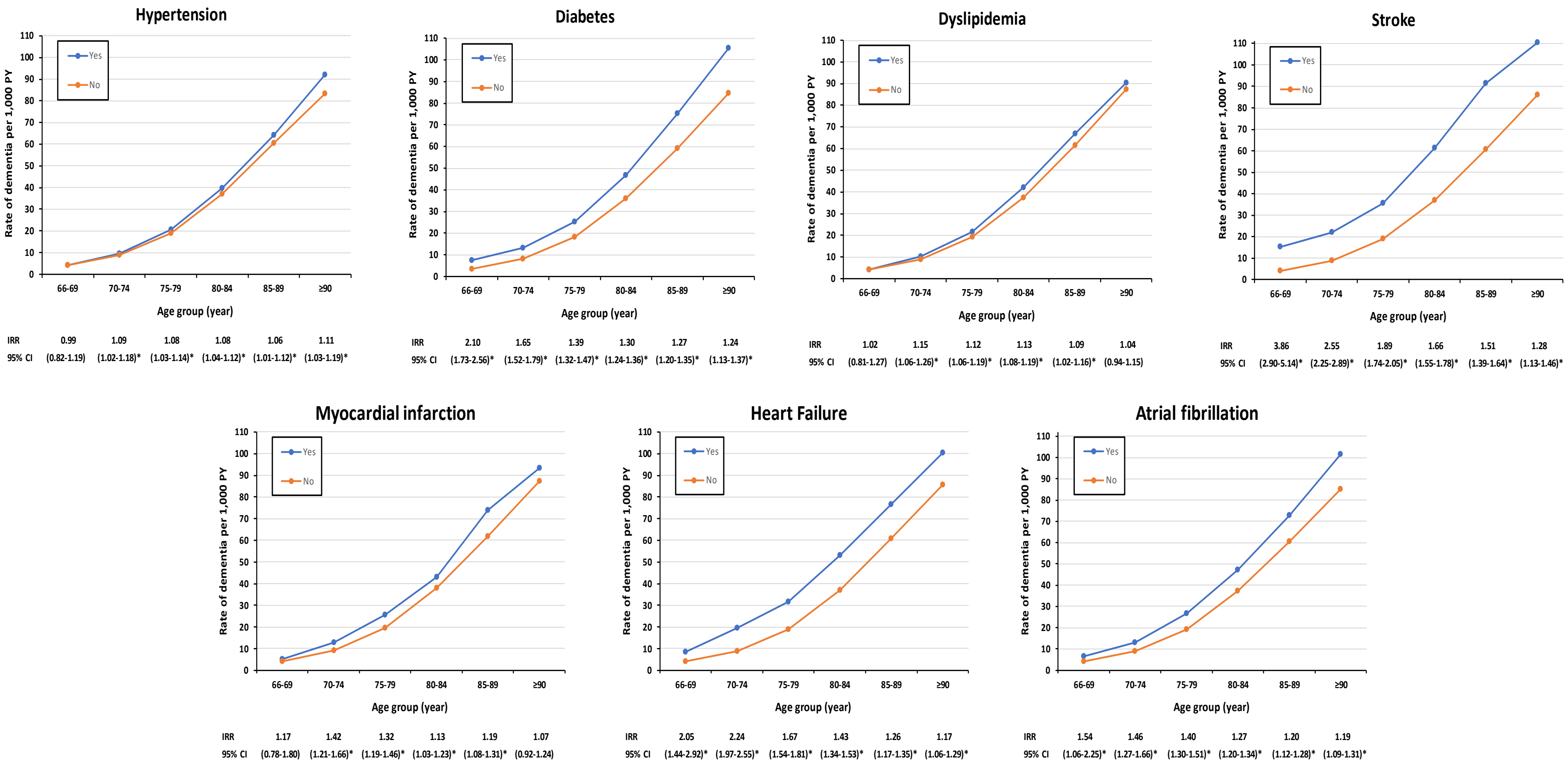

Figure S2b. Incidence Rate Ratios (IRR) of dementia in men per age group according to cardiovascular risk factors or disease per age groups. *PY* person-years, *IRR* incident rate ratio, *CI* confidence interval, * $p < 0.05$.
